# Embryo Screening for Polygenic Disease Risk: Recent Advances and Ethical Considerations

**DOI:** 10.3390/genes12081105

**Published:** 2021-07-21

**Authors:** Laurent C. A. M. Tellier, Jennifer Eccles, Nathan R. Treff, Louis Lello, Simon Fishel, Stephen Hsu

**Affiliations:** 1 Department of Physics and Astronomy, Michigan State University, East Lansing, MI 48824, USA; laurent@genomicprediction.com (L.C.A.M.T.); hsu@msu.edu (S.H.); 2 Genomic Prediction, Inc., North Brunswick, NJ 08902, USA; jennifer@genomicprediction.com (J.E.); nathan@genomicprediction.com (N.R.T.); 3 CARE Fertility Group, Nottingham NG8 6PZ, UK; professorfishel@gmail.com; 4 School of Pharmacy and Biomolecular Sciences, Liverpool John Moores University, Liverpool L2 2QP, UK

**Keywords:** genomics, complex trait prediction, PRS, in vitro fertilization, genetic engineering

## Abstract

Machine learning methods applied to large genomic datasets (such as those used in GWAS) have led to the creation of polygenic risk scores (PRSs) that can be used identify individuals who are at highly elevated risk for important disease conditions, such as coronary artery disease (CAD), diabetes, hypertension, breast cancer, and many more. PRSs have been validated in large population groups across multiple continents and are under evaluation for widespread clinical use in adult health. It has been shown that PRSs can be used to identify which of two individuals is at a lower disease risk, even when these two individuals are siblings from a shared family environment. The relative risk reduction (RRR) from choosing an embryo with a lower PRS (with respect to one chosen at random) can be quantified by using these sibling results. New technology for precise embryo genotyping allows more sophisticated preimplantation ranking with better results than the current method of selection that is based on morphology. We review the advances described above and discuss related ethical considerations.

## 1. Introduction

Over a million babies are born each year via IVF [1,2]. It is not uncommon for IVF parents to have more than one viable embryo from which to choose, as typical IVF cycles can produce four or five. The embryo that is transferred may become their child, while the others might not be used at all. We refer to this selection problem as the “embryo choice problem”. In the past, selections were made based on criteria such as morphology (i.e., rate of development, symmetry, general appearance) and chromosomal normality as determined by aneuploidy testing.

Recently, large datasets of human genomes together with health and disease histories have become available to researchers in computational genomics [3]. Statistical methods from machine learning have allowed researchers to build risk predictors (e.g., for specific disease conditions or related quantitative traits, such as height or longevity) that use the genotype alone as input information. Combined with the precision genotyping of embryos, these advances provide significantly more information that can be used for embryo selection to IVF parents.

In this brief article, we provide an overview of the advances in genotyping and computational genomics that have been applied to embryo selection. We also discuss related ethical issues, although a full discussion of these would require a much longer paper. Indeed, an in-depth review was recently published by Professor Julian Savulescu—Director of the Oxford Uehiro Centre for Practical Ethics—and collaborators [4], which we discuss below. Our purpose is to make bioethicists and philosophers more aware of recent scientific and technological breakthroughs (i.e., the current research frontier and state of the art), as well as to inform medical genomics and IVF researchers of some ethical perspectives. No attempt at an entirely comprehensive treatment of either scientific or ethical issues is contemplated, but we hope to further a well-informed discussion in this important area.

## 2. Polygenic Risk Scores (PRSs)

**Figure 1:**
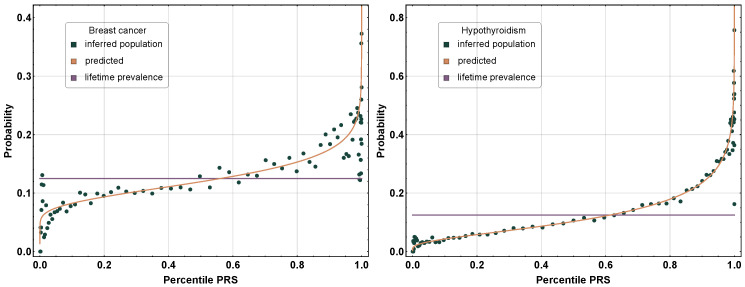
Incidence of breast cancer and hypothyroidism as a function of the polygenic risk score (PRS) percentile. At a high PRS, the likelihood of incidence increases nonlinearly, and at a low PRS, the likelihood decreases nonlinearly. The red curve indicates the theoretical, modeling case, and control populations with normal distributions that were shifted in the mean PRS. The blue data points were calculated using individuals (not used in training) binned by the PRSs. Reproduced from [5].

Polygenic risk predictors for dozens of important disease conditions have been published and validated by numerous research groups around the world [5,6,7,8,9,10,11,12]. We can roughly characterize the performance of these polygenic risk predictors as follows: Individuals with very high PRSs will typically have an incidence rate that is many times higher than the population average. For example, in [5], it was found that for atrial fibrillation, a 99th percentile PRS implies ∼10 times higher likelihood of case status. The rapid, nonlinear increase in absolute risk for the condition with the PRS percentile is shown in Figure 1 below. For outliers at very high PRS percentiles (e.g., within the top 1%), risk can exceed that associated with well-known monogenic risk factors, such as BRCA1 and BRCA2 [7]. Absolute risk can even approach 1 (near certainty) for some individuals.

There are now many validations of polygenic prediction in the scientific literature, which were conducted using groups of people born on different continents and in different decades with respect to the original populations used in training [10,13,14]. However, it is important to note that predictors work best when applied to ancestry groups that are similar to the original training population, and performance falls off with genetic distance [11,15]. It has also been shown that predictors can differentiate between siblings—for example, determining which one of them will experience a heart attack—despite similarity in childhood environments and genotype. The predictors work almost as well in pairwise sibling comparisons as in comparisons between randomly selected strangers [16].

Given one sibling with a normal-range PRS (less than the 84th percentile) and one sibling with a high PRS (e.g., the top 5 percentile, see [16] ), the predictors identify the affected sibling in about 70–90 percent of the cases across a variety of disease conditions, including breast cancer, heart attack, type 2 diabetes, and schizophrenia. For height, the predictor correctly identifies the taller sibling in roughly 80 percent of the cases when the (male) height difference is 2 inches or more [17].

There is already significant research on the application of PRSs in a clinical setting [5,8,12,18,19,20,21,22,23,24,25,26]. As a concrete example, women with high PRSs for breast cancer can be offered early screening—this is already the standard of care for those with BRCA risk variants [27,28]. However, BRCA mutations affect no more than a few women per thousand in the general population [29,30,31]. Importantly, the number of (BRCA-variant negative) women who are at high risk for breast cancer due to polygenic effects is an order of magnitude larger than the population of BRCA-variant carriers [5,7,32,33,34]. Precision genetics are already used in the identification of candidates for early intervention and will become widespread in the near future (cf. Myriad’s riskScore test and other examples [33,34]).

## 3. Precision Embryo Genotyping

Embryo biopsies (typically 3–7 cells) contain only a small amount of DNA, so it is a challenging problem to obtain accurate genotypes from them [35]. The problem is ameliorated by the widespread use of embryo freezing in IVF (in the past fresh embryo transfer required short turnaround times for genotyping results [36]), but amplification of small amounts of DNA still presents challenges for accurate genotyping. This problem has been solved by genomic prediction (GP) [35], thus allowing the application of PRSs in IVF. The GP process uses parental genotypes and the genotypes of other embryos (siblings) to perform error correction, achieving genotyping accuracy exceeding even that of clinical saliva genotyping on similar hardware platforms (99.6%), which is sufficient to accurately evaluate PRSs. This highly customized bioinformatics pipeline enables not only reliable polygenic disease prediction, but also other applications that rely on genotyping, such as fingerprinting, allelic ratio determination, polyploidy detection, relatedness QC checks, and contamination QC checks—resulting in a far superior performance in basic PGT (preimplantation genetic testing). A 99.6% genotyping accuracy means that the same sample genotyped twice will give the same clinical result twice, in contrast with noisier methods, such as traditional NGS (next-generation sequencing).

Carmi et al. [37] recently obtained estimates of risk reduction resulting from embryo selection using PRSs. For example, a relative risk reduction of ≈50% for schizophrenia could be achieved by selecting the embryo with the lowest PRS out of five viable embryos. While prevalence of schizophrenia is only roughly 1% in the general population, among families with a history of the condition, it is 11%. Therefore, the risk reduction can be large both in relative and absolute terms (i.e., conditional on family history).

Turley et al. [38] have also computed risk reductions for a variety of conditions, such as hypertension, type 2 diabetes, and coronary artery disease. Their results are broadly consistent with earlier results using sibling data [16]. They find somewhat smaller (but still beneficial) risk reductions in the case of non-European ancestry embryos.

## 4. Ethical Considerations

The results in the previous sections strongly support the claim that use of these methods in embryo screening reduces the risk of common disease conditions. On this basis alone, a utilitarian argument can be made for PRSs in IVF.

For further clarification, we explore a specific scenario involving breast cancer. It is well known that monogenic BRCA1 and BRCA2 variants predispose women to breast cancer, but this population is small—perhaps a few per thousand in the general population. The subset of women who do not carry a BRCA1 or BRCA2 risk variant but are at high polygenic risk is about ten times as large as the BRCA1/2 group. Thus, the majority of breast cancer can be traced to polygenic causes in comparison with commonly tested monogenic variants.

For BRCA carrier families, preimplantation screening against BRCA is a standard (and largely uncontroversial) recommendation [39]. The new technologies discussed here allow a similar course of action for the much larger set of families with breast cancer history who are not carriers of BRCA1 or BRCA2. They can screen their embryos in favor of a daughter whose breast cancer PRS is in the normal range, avoiding a potentially much higher absolute risk of the condition.

The main difference between monogenic BRCA screening and the new PRS screening against breast cancer is that the latter technology can help an order of magnitude more families. From an ethical perspective, it would be unconscionable to deny PRS screening to BRCA1/2-negative families with a history of breast cancer.

We believe that almost identical arguments apply to PRS screening for many other important disease conditions (e.g., type 1/2 diabetes or schizophrenia).

We are aware that this novel technology will reveal potential ethical challenges for some. Medical involvement in human procreation, especially since the conceptualization of IVF as a clinical resolution to childlessness more than 50 years ago, has presented constant ethical debates throughout its evolution. Indeed, some of the technologies that were deemed “ethically dubious” when first introduced (such as IVF itself, as well as aneuploidy screening) were, over time, incorporated into routine IVF practice. It is not within the ambit of this paper to provide the range of potential ethical deliberations for PGT-P, and this has been endeavored elsewhere [4]. However, it is important to recognize the pillars of medical ethics for the introduction of new technologies, especially those that can have ongoing generational impacts: autonomy, beneficence, non-maleficence, and justice.

PGT-P of preimplantation embryos is only available to couples who can afford to undertake IVF and genetic screening of their preimplantation embryos. Even in countries that provide a comprehensive national healthcare program, IVF began as a privately funded medical service, and this still predominates. For many couples, the right to choose exists, but only if they can afford many of the options open to them. In most societies, the opportunity to choose trumps the principle of justice, more through societal pragmatism than desire, thus raising concerns that we must strive to treat everyone alike, but genetic advantage is available only to the wealthy [40]. Not only does this issue arise in many aspects of human innovation, with, perhaps, the greatest reservations being directed at medically assisted procreation, but history also demonstrates that with the launching of such benefits on a small scale amidst a welter of debate (and sometimes outrage), over time, opportunity and acceptance widen across society.

It can be hard to argue against beneficence when human health is one of the main aspirations in the global effort to improve human well-being. Whilst acknowledging that polygenic scoring selects for health improvement in any single embryo over another, providing for an individual’s “healthspan” is, from the moment of birth, a desire of both parents and society alike. Similar deliberations are rationalized from other embryo screening options (such as aneuploidy or monogenic screening) to amniocentesis and beyond; for example, dietary control or acquiring the best education. Under the aegis of “do no harm”, some may argue that parent–child relationships may be affected by particular knowledge of the health score of the embryo. Careful consideration needs to be given to potential parental anxiety over a health score, balanced against the provision of such knowledge benefiting the family, such as knowing if there is an elevated risk of diabetes early on and moderating lifestyle appropriately. Indeed, this might sit well for those in favor of preventive and personalized medicine. We must then regard parental choice as an important aspiration, but each potential parent will need to be fully supported with comprehensive counseling, which itself has always been a cornerstone of IVF practice.

As already mentioned, we do not attempt a comprehensive discussion of all of the ethical issues raised by IVF polygenic screening. For that, we refer the reader to the recent article “Three models for the regulation of polygenic scores in reproduction” by Munday and Savalescu [4]. An incomplete list of the topics investigated there includes the impact of PGT-P on inequality, selection on non-medical traits, such as cosmetic traits or cognitive ability, impact on genetic diversity, effect on parent–child relationships, and potential regulatory structures. Munday and Savalescu, as philosophers, locate their analysis within distinct frameworks that adopt specific ethical priors (about which reasonable people might disagree): a Welfarist model, a Libertarian model, the Expressivist critique, etc. For example, their Welfarist model prioritizes the well-being of the resulting child in embryo selection. The conclusions reached depend on individual choices concerning distinct values and principles. Obviously, these considerations are both complex and subtle. We will not do them justice here.

The arguments given above notwithstanding, individual physicians are entitled to their own judgement regarding new technologies. The American Medical Association recommends the following: In general, physicians should refer a patient to another physician or institution to provide treatment the physician declines to offer. When a deeply held, well-considered personal belief leads a physician also to decline to refer, the physician should offer impartial guidance to patients about how to inform themselves regarding access to desired services. (AMA Physician Exercise of Conscience: Code of Medical Ethics Opinion 1.1.7 [41].)

## 5. Clinical Application of the Embryo Health Score

**Figure 2:**
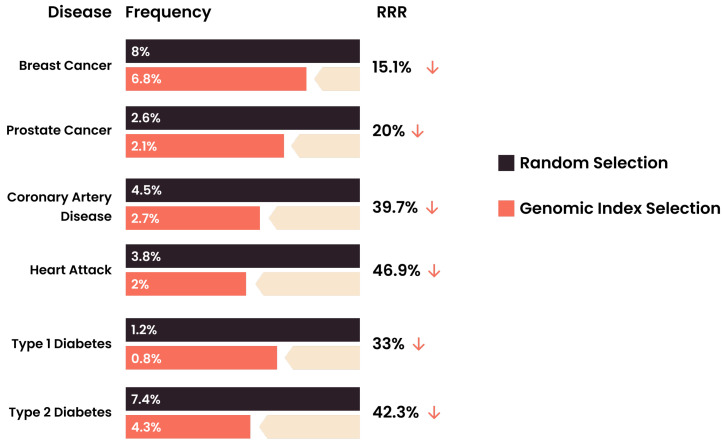
Relative risk reduction (RRR) from the use of the genomic index for transfer prioritization in the minimal case of prioritization between two euploid sibling embryos. The results were obtained from calculations on 11,000 actual sibling pairs to quantify how much less likely the sibling with lower polygenic risk was to have the condition [44].

For practical reasons, IVF physicians, genetic counselors, and patients require a uni-dimensional ranking metric, which we refer to here as an “Embryo Health Score” (EHS) (described elsewhere in the genomics literature as a “genomic index”). The EHS aggregates information from multiple risk scores and factors into a single number [42,43], which can be used as a tool for providing clear reproductive decision-making guidance to clinicians and families. Roughly speaking, the EHS is the sum of the predicted absolute risks for each disease condition weighted by the life-span impact of the condition (this life-span impact is taken directly from the existing medical literature). Guidance is provided via a proposed rank ordering of the embryos along this single EHS dimension. It has been demonstrated [44] that the EHS rank ordering can achieve significant risk reduction across a panel of important disease conditions (Figure 2). In the study cited above, the specific conditions used in the index were breast cancer, prostate cancer, testicular cancer, basal cell carcinoma, malignant melanoma, coronary artery disease, high cholesterol, hypertension, heart attack risk, type 1 diabetes, and type 2 diabetes. This list of conditions is by no means exhaustive or necessarily optimal; the quality of specific predictors is rapidly improving, the number of conditions for which good predictors exist is expanding, and finally, specific optimization targets, such as longevity, quality of life, cardiovascular health, etc., could affect the inclusion and/or relative weightings of predictors used in the index.

**Figure 3:**
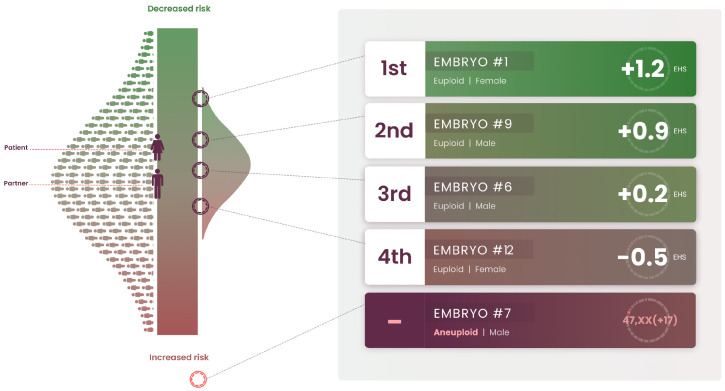
Sample EHS report that indicates the scores of the mother, father, and five embryos. The bell-shaped distribution on the right helps to visualize the distribution of the EHS that would result if the mother and father had a large number of children (the distribution on the left is for the general population). The five embryos can be compared to this (potential) distribution. One of the embryos is aneuploid. The data in this report were drawn from an actual case.

This validation of the genomic index addresses concerns arising from pleiotropy: that selection against one disease will generally prove to be selecting in favor of another. In fact, it is found that selection using EHS simultaneously reduces the risk across the entire panel of disease conditions; see Figure 2 and Figure 3. This result is unsurprising given that the degree of pleiotropy between polygenic risk predictors is modest; regions of the genome that are used to predict a specific disease risk A generally have modest overlap with regions that predict a specific risk B (e.g., disease A could be diabetes and B could be hypothyroidism). This genetic architecture was analyzed in detail in [45]. Thus, it is possible for an individual to be low risk across a large number of disease conditions simultaneously, and a genetic index of the kind described above helps to identify such cases.

Before 2019, preimplantation genetic testing (PGT) was confined to specific rare variants of large effect. Now, it is possible to amalgamate all factors—rare variants of large effect, such as BRCA1, thousands of smaller-effect SNPs, copy number variations, and even biomarkers—into a single test, which can inform the decision as to which embryo to prioritize.

## 6. Discussion and Conclusions

We have described three new technologies that are already making a significant impact on assisted human reproduction (IVF): polygenic risk scores, precision genotyping of embryos, and genomic indices that can predict overall health or even longevity. These technologies are likely to dramatically advance and achieve broad utilization in the coming years. A strong case can be made that patients will benefit significantly from these developments, but the associated ethical issues deserve serious attention, as exemplified in the work of Professor Julian Savulescu—Director of the Oxford Uehiro Centre for Practical Ethics—and collaborators [4]. We hope that this article is the beginning of a far-reaching conversation between scientists, ethicists, medical professionals, and broader society.

## Data Availability

Not applicable.

## References

[1] Fauser B.C. (2019). Towards the global coverage of a unified registry of IVF outcomes. Reprod. Biomed. Online.

[2] European Society of Human Reproduction and Embryology ART Fact Sheet. https://www.eshre.eu/-/media/sitecore-files/Press-room/ART-fact-sheet-2020-data-2016.pdf?la=en&hash=AB68A67B4FEA7723F2125B02BCB93FB837139CD4.

[3] Raben T.G., Lello L., Widen E., Hsu S.D.H. (2021). From Genotype to Phenotype: Polygenic Prediction of Complex Human Traits. http://xxx.lanl.gov/abs/2101.05870.

[4] Munday S., Savulescu J. (2021). Three models for the regulation of polygenic scores in reproduction. J. Med. Ethics.

[5] Lello L., Raben T.G., Yong S.Y., Tellier L.C., Hsu S.D.H. (2019). Genomic prediction of 16 complex disease risks including heart attack, diabetes, breast and prostate cancer. Sci. Rep..

[6] Khera A.V., Chaffin M., Aragam K.G., Haas M.E., Roselli C., Choi S.H., Natarajan P., Lander E.S., Lubitz S.A., Ellinor P.T. (2018). Genome-wide polygenic scores for common diseases identify individuals with risk equivalent to monogenic mutations. Nat. Genet..

[7] Khera A.V., Chaffin M., Wade K.H., Zahid S., Brancale J., Xia R., Distefano M., Senol-Cosar O., Haas M.E., Bick A. (2019). Polygenic prediction of weight and obesity trajectories from birth to adulthood. Cell.

[8] Lewis C.M., Vassos E. (2017). Prospects for using risk scores in polygenic medicine. Genome Med..

[9] Lambert S.A., Gil L., Jupp S., Ritchie S.C., Xu Y., Buniello A., Abraham G., Chapman M., Parkinson H., Danesh J. (2020). The Polygenic Score Catalog: An open database for reproducibility and systematic evaluation. medRxiv.

[10] Wünnemann F., Sin Lo K., Langford-Avelar A., Busseuil D., Dubé M.P., Tardif J.C., Lettre G. (2019). Validation of Genome-Wide Polygenic Risk Scores for Coronary Artery Disease in French Canadians. Circ. Genom. Precis. Med..

[11] Privé F., Aschard H., Carmi S., Folkersen L., Hoggart C., O’Reilly P.F., Vilhjálmsson B.J. (2021). High-resolution portability of 245 polygenic scores when derived and applied in the same cohort. medRxiv.

[12] Torkamani A., Wineinger N.E., Topol E.J. (2018). The personal and clinical utility of polygenic risk scores. Nat. Rev. Genet..

[13] Belsky D.W., Domingue B.W., Wedow R., Arseneault L., Boardman J.D., Caspi A., Conley D., Fletcher J.M., Freese J., Herd P. (2018). Genetic analysis of social-class mobility in five longitudinal studies. Proc. Natl. Acad. Sci. USA.

[14] Mars N., Koskela J.T., Ripatti P., Kiiskinen T.T.J., Havulinna A.S., Lindbohm J.V., Ahola-Olli A., Kurki M., Karjalainen J., Palta P. (2020). Polygenic and clinical risk scores and their impact on age at onset and prediction of cardiometabolic diseases and common cancers. Nat. Med..

[15] Durvasula A., Lohmueller K.E. (2021). Negative selection on complex traits limits phenotype prediction accuracy between populations. Am. J. Hum. Genet..

[16] Lello L., Raben T.G., Hsu S.D.H. (2020). Sibling validation of polygenic risk scores and complex trait prediction. Sci. Rep..

[17] Lello L., Avery S.G., Tellier L., Vazquez A.I., de los Campos G., Hsu S.D. (2018). Accurate genomic prediction of human height. Genetics.

[18] Liu L., Kiryluk K. (2018). Genome-wide polygenic risk predictors for kidney disease. Nat. Rev. Nephrol..

[19] Chatterjee N., Shi J., García-Closas M. (2016). Developing and evaluating polygenic risk prediction models for stratified disease prevention. Nat. Rev. Genet..

[20] Euesden J., Lewis C.M., O’reilly P.F. (2014). PRSice: Polygenic risk score software. Bioinformatics.

[21] Shieh Y., Shieh Y., Hu D., Ma L., Huntsman S., Gard C.C., Leung J.W.T., Tice J.A., Vachon C.M., Cummings S.R. (2016). Breast cancer risk prediction using a clinical risk model and polygenic risk score. Breast Cancer Res. Treat..

[22] Abraham G., Inouye M. (2015). Genomic risk prediction of complex human disease and its clinical application. Curr. Opin. Genet. Dev..

[23] Priest J.R., Ashley E.A. (2014). Genomics in clinical practice. BMJ Heart.

[24] Jacob H.J., Abrams K., Bick D.P., Brodie K., Dimmock D.P., Farrell M., Geurts J., Harris J., Helbling D., Joers B.J. (2013). Genomics in clinical practice: Lessons from the front lines. Sci. Transl. Med..

[25] Veenstra D.L., Roth J.A., Garrison L.P., Ramsey S.D., Burke W. (2010). A formal risk-benefit framework for genomic tests: Facilitating the appropriate translation of genomics into clinical practice. Genet. Med..

[26] Bowdin S., Gilbert A., Bedoukian E., Carew C., Adam M.P., Belmont J., Bernhardt B., Biesecker L., Bjornsson H.T., Blitzer M. (2016). Recommendations for the integration of genomics into clinical practice. Genet. Med..

[27] Nelson H.D., Pappas M., Cantor A., Haney E., Holmes R. (2019). Risk assessment, genetic counseling, and genetic testing for BRCA-related cancer in women: Updated evidence report and systematic review for the US Preventive Services Task Force. JAMA.

[28] Amir E., Freedman O.C., Seruga B., Evans D.G. (2010). Assessing women at high risk of breast cancer: A review of risk assessment models. J. Natl. Cancer Inst..

[29] Offit K. (2006). BRCA Mutation Frequency and Penetrance: New Data, Old Debate. J. Natl. Cancer Inst..

[30] Ford D., Easton D.F., Peto J. (1995). Estimates of the gene frequency of BRCA1 and its contribution to breast and ovarian cancer incidence. Am. J. Hum. Genet..

[31] Whittemore A.S., Gong G., John E.M., McGuire V., Li F.P., Ostrow K.L., Dicioccio R., Felberg A., West D.W. (2004). Prevalence of BRCA1 mutation carriers among U.S. non-Hispanic Whites. Cancer Epidemoiol. Biomark. Prev..

[32] Kuchenbaecker K., McGuffog L., Barrowdale D., Lee A., Soucy P., Dennis J., Domchek S.M., Robson M., Spurdle A.B., Ramus S.J. (2017). Evaluation of Polygenic Risk Scores for Breast and Ovarian Cancer Risk Prediction in BRCA1 and BRCA2 Mutation Carriers. J. Natl. Cancer Inst..

[33] Hughes E., Tshiaba P., Gallagher S., Wagner S., Judkins T., Roa B., Rosenthal E., Domchek S., Garber J., Lancaster J. (2020). Development and Validation of a Clinical Polygenic Risk Score to Predict Breast Cancer Risk. JCO Precis. Oncol..

[34] (2020). Myriad—Home. https://www.myriadmyrisk.com.

[35] Treff N.R., Zimmerman R., Bechor E., Hsu J., Rana B., Jensen J., Li J., Samoilenko A., Mowrey W., Alstine J.V. (2019). Validation of concurrent preimplantation genetic testing for polygenic and monogenic disorders, structural rearrangements and whole and segmental chromosome aneuploidy with a single universal platform. Eur. J. Med. Genet..

[36] Kumar A., Ryan A., Kitzman J.O., Wemmer N., Snyder M.W., Sigurjonsson S., Lee C., Banjevic M., Zarutskie P.W., Lewis A.P. (2015). Whole genome prediction for preimplantation genetic diagnosis. Genome Med..

[37] Lencz T., Backenroth D., Granot-Hershkovitz E., Green A., Gettler K., Cho J.H., Weissbrod O., Zuk O., Carmi S. (2021). Utility of polygenic embryo screening for disease depends on the selection strategy. bioRxiv.

[38] Turley P., Meyer M., Wang N., Cesarini D., Hammonds E., Martin A., Neale B., Rehm H., Wilkins-Haug L., Benjamin D. (2021). Problems with Using Polygenic Scores to Select Embryos. N. Engl. J. Med..

[39] Ethics Committee of the American Society for Reproductive Medicine (2018). Use of preimplantation genetic testing for monogenic defects (PGT-M) for adult-onset conditions: An Ethics Committee opinion. Fertil. Steril..

[40] Singer P., Savulescu J., Bostrom N. (2009). Parental choice and human development. Human Enhancement.

[41] Physician Exercise of Conscience—Code of Medical Ethics Opinion 1.1.7. https://journalofethics.ama-assn.org/article/ama-code-medical-ethics-opinions-related-moral-distress/2017-06.

[42] Timmers P.R., Mounier N., Läll K., Fischer K., Ning Z., Feng X., Bretherick A., Clark D.W., Consortium E., Shen X. (2018). Genomic underpinnings of lifespan allow prediction and reveal basis in modern risks. bioRxiv.

[43] Meisner A., Kundu P., Zhang Y.D., Lan L.V., Kim S., Ghandwani D., Pal Choudhury P., Berndt S.I., Freedman N.D., Garcia-Closas M. (2020). Combined Utility of 25 Disease and Risk Factor Polygenic Risk Scores for Stratifying Risk of All-Cause Mortality. Am. J. Hum. Genet..

[44] Treff N.R., Eccles J., Marin D., Messick E., Lello L., Gerber J., Xu J., Tellier L.C. (2020). Preimplantation genetic testing for polygenic disease relative risk reduction: Evaluation of genomic index performance in 11,883 adult sibling pairs. Genes.

[45] Yong S.Y., Raben T.G., Lello L., Hsu S.D. (2020). Genetic Architecture of Complex Traits and Disease Risk Predictors. Sci. Rep..

